# Applying a genetic risk score for prostate cancer to men with lower urinary tract symptoms in primary care to predict prostate cancer diagnosis: a cohort study in the UK Biobank

**DOI:** 10.1038/s41416-022-01918-z

**Published:** 2022-08-18

**Authors:** Harry D. Green, Samuel W. D. Merriel, Richard A. Oram, Katherine S. Ruth, Jessica Tyrrell, Samuel E. Jones, Chrissie Thirlwell, Michael N. Weedon, Sarah E. R. Bailey

**Affiliations:** 1 Exeter Centre of Excellence for Diabetes Research (EXCEED), University of Exeter Medical School, St Luke’s Campus, University of Exeter, Heavitree Road, Exeter, Devon EX1 2LU UK; 2 DISCOVERY Group, University of Exeter Medical School, St Luke’s Campus, University of Exeter, Heavitree Road, Exeter, Devon EX1 2LU UK; 3 Institute of Biomedical and Clinical Science, University of Exeter Medical School, St Luke’s Campus, University of Exeter, Heavitree Road, Exeter, Devon EX1 2LU UK; 4 Genetics of Complex Traits, University of Exeter Medical School, University of Exeter, Exeter, EX2 5DW UK; 5 Institute for Molecular Medicine (FIMM), University of Helsinki, Helsinki, Finland; 6 University of Exeter Medical School, St Luke’s Campus, University of Exeter, Heavitree Road, Exeter, Devon EX1 2LU UK; 7 UCL Cancer Institute, Huntley St, London, EX1 2LU UK

**Keywords:** Risk factors, Diagnostic markers, Cancer genomics, Prostate cancer, Diagnosis

## Abstract

**Background:**

Prostate cancer is highly heritable, with >250 common variants associated in genome-wide association studies. It commonly presents with non-specific lower urinary tract symptoms that are frequently associated with benign conditions.

**Methods:**

Cohort study using UK Biobank data linked to primary care records. Participants were men with a record showing a general practice consultation for a lower urinary tract symptom. The outcome measure was prostate cancer diagnosis within 2 years of consultation. The predictor was a genetic risk score of 269 genetic variants for prostate cancer.

**Results:**

A genetic risk score (GRS) is associated with prostate cancer in symptomatic men (OR per SD increase = 2.12 [1.86–2.41] *P* = 3.5e-30). An integrated risk model including age and GRS applied to symptomatic men predicted prostate cancer (AUC 0.768 [0.739–0.796]). Prostate cancer incidence was 8.1% (6.7–9.7) in the highest risk quintile. In the lowest quintile, prostate cancer incidence was <1%.

**Conclusions:**

This study is the first to apply GRS in primary care to improve the triage of symptomatic patients. Men with the lowest genetic risk of developing prostate cancer could safely avoid invasive investigation, whilst those identified with the greatest risk could be fast-tracked for further investigation. These results show that a GRS has potential application to improve the diagnostic pathway of symptomatic patients in primary care.

## Introduction

Prostate cancer accounts for around a quarter of new cancer cases in men, approximately 52,000 per year in the UK, and is increasing by around 4% annually [1]. An estimated 14% of prostate cancer deaths in the UK could be avoided with earlier detection [2]; advanced stage at diagnosis is associated with poorer survival [3]. Most men with prostate cancer are diagnosed after attending primary care with symptoms [4]. Evidence on the benefit of prostate cancer screening programmes (targeting asymptomatic men) is mixed; a large European screening trial identified significant reductions in prostate cancer mortality [5], while other have found that increases in prostate cancer incidence associated with screening trials were not accompanied by significant decreases in mortality [6, 7], suggesting possible overdiagnosis [8].

Lower urinary tract symptoms (LUTS), such as nocturia, urinary frequency or poor stream, are common in men aged 50 and above, and are often present at the time of a prostate cancer diagnosis. The incidence of LUTS, benign prostate enlargement, and prostate cancer all rise with increasing age, complicating attempts to accurately diagnose tumours. The evidence for an association between LUTS and the risk of prostate cancer is equivocal [9], and very few studies have assessed this association in a primary care population [10].

The UK’s National Institute for Health and Care Excellence (NICE) recommends a prostate-specific antigen (PSA) test for men in primary care with LUTS or new onset erectile dysfunction [11]. PSA is the only test currently available for detecting prostate cancer in primary care, yet the diagnostic accuracy of PSA in symptomatic men is unclear [9]. The most recent systematic review of the diagnostic accuracy of PSA for prostate cancer in patients with LUTS found that a PSA threshold of 4 ng/mL had a sensitivity of 0.93 (95% CI 0.88, 0.96) and specificity of 0.20 (95% CI 0.12, 0.33), and the area under the curve (AUC) was 0.72 (95% CI 0.68, 0.76) [12]. All included studies for the review were conducted in secondary care patient cohorts, limiting the applicability of the findings to the primary care setting, where cancer incidence is lower, and therefore AUC likely to be lower, due to spectrum bias [13]. As the studies in that review were based on observational data, ascertainment bias and lack of follow-up in PSA-negative men may mean that the true AUC of PSA in symptomatic men in primary care is lower still.

Over the past 15 years, genome-wide association studies (GWAS) have identified over 250 individual genetic variants that contribute to the development of prostate cancer, which have been combined into a clinically useful measure that reflects an individual’s risk of developing prostate cancer: a genetic risk score (GRS) [14]. GRS improve risk predictions based on family history alone [15–17] but despite promising evidence on predictive ability, there has been limited integration of GRS into clinical practice [18]. There are no studies of the application of a prostate cancer GRS in the targeted investigation of men with LUTS. It is not known whether the genetic risk of developing prostate cancer affects the chance of it being present in symptomatic men, or whether GRS could be helpful in selecting men for further investigation once they present with LUTS.

The objective of this study is to assess if a prostate cancer GRS predicted a new diagnosis of prostate cancer in men in the UK Biobank who consulted their general practitioner (GP) with LUTS.

## Methods

### Public and patient involvement

An existing patient and public involvement and engagement (PPI&E) group consisting of six men with personal experience of prostate cancer investigation informing on-going prostate cancer research at the University of Exeter was involved with the development of the research question for this study. Their views were specifically sought around the acceptability of developing an integrated risk model that required the incorporation of genetic information, and the additional risk factors to consider. These men felt the potential benefits in improving early detection of prostate cancer and avoiding unnecessary, invasive diagnostic tests outweighed concerns about using genetic data. They also highlighted the importance of a patient’s age and family history in assessing prostate cancer risk.

### Participants

Unrelated UK Biobank participants of white European ancestry were included in this study. Principal component analysis was performed using individuals from the 1000 Genomes Project prior to the projection of UK Biobank individuals into the principal component space. K-means clustering was subsequently applied to classify individuals as European, with centres initiated to the mean principal component values of each 1000 Genomes sub-population. The first four principal components were used in this analysis. Related individuals were defined using a KING Kinship [19] to exclude those third-degree relatives or closer. An optimal list of unrelated individuals was generated by preferentially removing individuals with the maximum number of relatives to allow maximum numbers of individuals to be included; e.g. if A was related to B and C, but B and C were not, A was removed. For a simple pair, one individual was removed at random.

Participants were included in the analysis if they had any of these recorded in the UKBB GP records: incontinence, nocturia, hesitancy, frequency, urgency, retention, poor stream, double voiding, or a general code of lower urinary tract symptoms (LUTS). Read codes for each condition can be found in Supplementary Table 1. The date of the first relevant symptom on record was defined as the index date for each participant.

### Variable definition

Prostate cancer was defined using the earliest date of either the Read code ‘B46..’ in GP records, or the linked cancer registry data. As this study aimed to test the ability of a prostate cancer GRS to identify new prostate cancer in men with symptoms, patients with prostate cancer recorded prior to the index date were excluded. Patients in the symptomatic cohort that were diagnosed with prostate cancer within 2 years of the index date were treated as cases. Patients with no record of a prostate cancer diagnosis within 2 years of the index date were considered controls. Controls may have been diagnosed with prostate cancer more than 2 years after the index date; this follow-up period was selected so that only prostate cancers that could be causing symptoms were detected. These could be diagnosed at the time the patient is symptomatic. While there is no perfect cutoff date for this, 2 years is a commonly accepted limit in previous research in cancer diagnosis [10, 20–26].

A genetic risk score for prostate cancer was derived using the 269 known risk variants reported in a recent trans-ancestry genome-wide meta-analysis; the included variants are described in Conti et al. [14]. Weighting for each single nucleotide polymorphism (SNP) was given by the log of the European odds ratio from Supplementary Table 4 of Conti et al. These weights were used over the UK Biobank weights to avoid issues with overfitting. The GRS was calculated for each UK Biobank participant using the sum of the weights multiplied by the participant’s genotype.

Body mass index (BMI) was defined using UK Biobank’s Data-Field 21001 and reported as mean kg/m^2^, ± standard deviation. Smoking status (ever or never) was defined using Data-Field 1239. Family history of prostate cancer was defined using self-report data (Data-Field 20111). These were measured at baseline UKBB recruitment.

Only a small proportion of the cohort had a PSA test result on record, and these were abnormal; the AUC for PSA alone was >0.9 which is unrealistic compared to the literature and likely to be the result of ascertainment bias [12]. As PSA is part of the current diagnostic pathway to determine if a patient is investigated for prostate cancer, it has a direct causal effect on whether an individual will be diagnosed with prostate cancer independently of the test’s ability to predict that outcome. Any model of PSA and GRS in an observational study like UK Biobank will be significantly biased towards PSA; patients with a negative PSA test are not followed up and therefore unlikely to be diagnosed with prostate cancer, even if it exists. Therefore, this study compared the performance of a prostate cancer GRS to published reports of PSA diagnostic accuracy.

### Statistical methods

All analysis was conducted using R 4.0.3 “Bunny-Wunnies Freak Out”. The cohort characteristics were described and tests for associations performed with baseline variables: index age, family history, smoking status and BMI. The association between the GRS and a prostate cancer diagnosis within 2 years of symptoms was evaluated in a simple logistic regression model, and the odds ratio reported per standard deviation increase in GRS. We also evaluated the hazard ratio using a Cox Proportional Hazards model. Controls who died within the 2-year study period were excluded from the logistic regression model as it cannot be ascertained whether they would have remained cancer-free for 2 years.

An integrated risk model was developed by including all permutations of predictor variables that reached nominal significance (*P* < 0.05) plus symptoms in addition to the GRS to test if predictive power was enhanced in any combination. As some participants had multiple symptoms recorded at the index date, the symptom profile could not be considered a categorical variable, and was modelled by treating each symptom as its own binary variable. The receiver operating characteristic (ROC) area under the curve (AUC) was estimated with 95% confidence intervals (CIs) for each possible integrated risk model to measure overall diagnostic performance. Diagnostic performance was estimated for incidence thresholds of 1, 2, 3, 4 and 5%; 3% is the current NICE threshold for investigation in guidance NG12 [11], although a drop to 2% is under consideration [27]. Patients have reported that they would prefer to be investigated at risk thresholds as low as 1% [28]. The study was reported in line with STROBE guidelines [29].

### Preprint

A previous version of this manuscript was published as a preprint [30].

## Results

### Cohort description

**Fig. 1:**
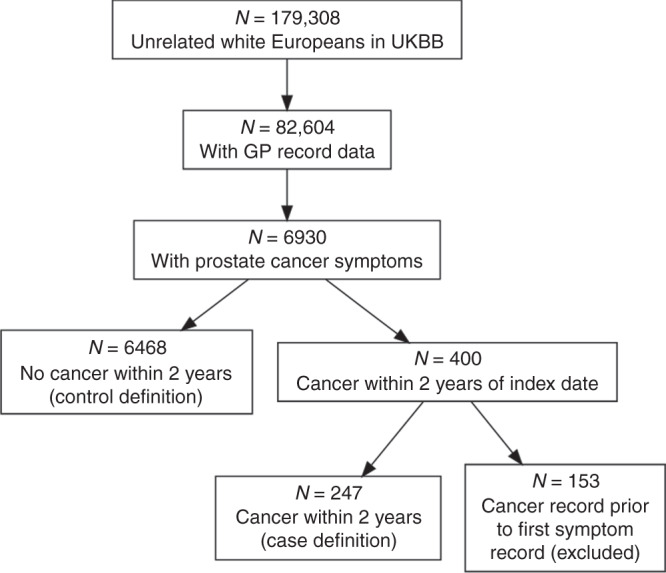
Patient selection flowchart. Flowchart showing how cases and controls were selected from the total population of unrelated white Europeans in UK Biobank. *N* = number.

Of the 179,308 unrelated white European men in UKBB, 82,604 had linked GP records, of which 6930 individuals reported relevant symptoms. 153 had evidence of prostate cancer prior to the first symptom report and were excluded. Of the 6777 without pre-existing prostate cancer, 247 had a record of prostate cancer within 2 years (3.5%) and were included as cases, of which 5 (2%) died during the 2-year period. Of the remaining 6530, 62 (0.9%) died during the 2-year follow-up and were excluded from case-control analyses, leaving 6468 controls. 3.7% of those included in the model were cases. Over 75% of the cohort were included following reports of LUTS, nocturia or frequency (Supplementary Table 2). Figure 1 shows how the case and control numbers were obtained. Over 75% of the cohort were included following reports of LUTS, nocturia or frequency (Supplementary Table 2).

Those who went on to develop prostate cancer tended to be older, but no other covariates were significantly associated at *P* < 0.05 (Table 1).

**Table 1:** Observational associations between cases and controls, estimated with logistic regression.

Variable	Cases	Controls	Odds ratio	*P* value
BMI	28.02 + /−3.9	28.06 + /−4.28	1.00 (0.97–1.03)	0.89
Index age	64.51 + /−5.95	59.54 + /−8.11	1.09 (1.07–1.11)	2.9e-20
Current smoker	21 (8.57%)	503 (7.89%)	1.09 (0.69–1.73)	0.70
Ever smoker	142 (57.96%)	3370 (52.88%)	1.23 (0.95–1.59)	0.12
Family history	25 (10.12%)	477 (7.37%)	1.41 (0.93–2.16)	0.11

*BMI* body mass index.

### A GRS predicts prostate cancer in men with symptoms

In men with symptoms, the prostate cancer genetic risk score was associated with the development of prostate cancer within the next 2 years. In the 247 men with a prostate cancer diagnosis within 2 years of symptoms, the mean GRS was 23.52 (SD 0.81) vs 22.92 (SD 0.79) in the 6468 men who were not diagnosed with prostate cancer (OR = 2.12 [1.86–2.41] *P* = 3.5e-30) per SD increase in GRS. Supplementary Fig. 1 shows the distributions of genetic risk score in men who were diagnosed with cancer within 2 years of symptom onset vs those who were not.

**Fig. 2:**
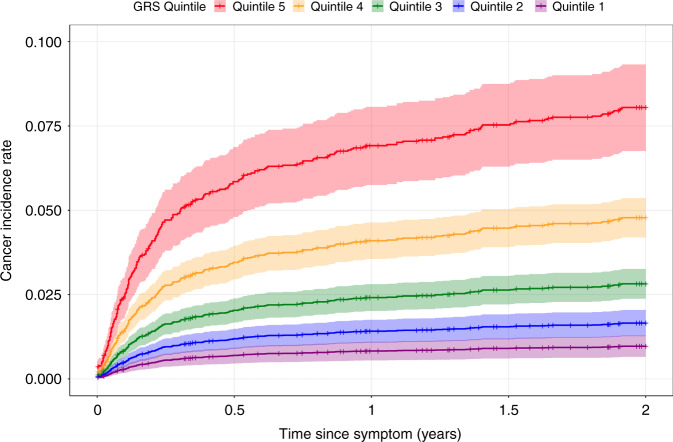
Cumulative hazard plot for prostate cancer. Cumulative hazard plot showing prostate cancer incidence rates over time from symptomatic presentation, stratified by genetic risk score quintile.

Prostate cancer incidence rate over time, stratified by GRS quintile, is shown in Fig. 2. Individuals with relevant symptoms who were in the lowest quintile of the GRS had an 8.8% (7.3–10%) chance to develop prostate cancer by the end of the 2-year period, while individuals in the top quintile had an 1% (0.59–1.8%) chance. Using Cox-PH modelling, the GRS had a hazard ratio of 2.06 (1.82–2.33), *P* = 1.5e-31 per SD increase in GRS.

### An integrated risk model of GRS and age has predictive power over and above GRS alone

An integrated risk model including GRS and age returned a ROC AUC of 0.772 (95% CI 0.744–0.8) (ROC curve shown in Supplementary Fig. 2). This was substantially stronger than either of the two individual covariates (GRS AUC: 0.703 [95% CI 0.67–0.736] and age AUC: 0.68 [95% CI 0.65–0.709]). Adding family history and symptom profile to the integrated risk model provided a negligible increase in predictive power (AUC: 0.782 [95% CI 0.755–0.81], Supplementary Table 3 [AUCs and 95% CIs of all permutations of GRS, age, family history and symptom profile]).

Predicted probability of 2-year prostate cancer incidence and diagnostic accuracy statistics are reported in Table 2 at thresholds of 1, 2, 3, 4 and 5%, in addition to the probability threshold that maximises Youden’s J statistic (3.7%). The integrated risk model had a negative predictive value of greater than 99% for thresholds of 0.02 or less.

**Table 2:** Diagnostic statistics estimated for risk thresholds of 1, 2, 3, 4 and 5%, plus the optimum threshold of 3.7% recommended by the model.

Threshold	Specificity	Sensitivity	PPV	NPV	Youden’s J
0.010	0.234	0.972	0.046	0.995	0.206
0.020	0.468	0.895	0.060	0.991	0.363
0.030	0.613	0.777	0.071	0.986	0.390
0.037	0.688	0.713	0.080	0.984	0.400
0.040	0.712	0.680	0.083	0.983	0.393
0.050	0.780	0.587	0.092	0.980	0.367

*PPV* positive predictive value, *NPV* negative predictive value.

In Table 3, the 2-year incidence rates of prostate cancer are stratified by age decade and GRS quintile. An incidence of <1% was observed in those aged 40 years and under in the bottom four GRS quintiles and aged 40–50 years in the bottom two GRS quintiles. Men aged 70 years and over had a >1% incidence rate in every GRS quintile, while men over 60 in the top GRS quintile had a >10% incidence rate.

**Table 3:** Two-year incidence rates of prostate cancer, broken down by age decade at time of symptom and GRS quintile.

GRS quintile	Under 50 (*n* = 911)	50–59 (*n* = 2321)	60–69 (*n* = 2816)	70 and over (*n* = 667)
First	0% (0–2.7%)	0.9% (0.29–2.4%)	1.2% (0.52–2.5%)	2.3% (0.58–7%)
Second	0% (0–2.4%)	0.87% (0.28–2.4%)	2.5% (1.4–4.2%)	4.7% (1.9–10%)
Third	0% (0–2.6%)	1.9% (0.95–3.8%)	4.5% (3–6.6%)	6% (2.8–12%)
Fourth	0% (0–2.8%)	1.8% (0.83–3.6%)	5.5% (3.9–7.8%)	6.1% (3–12%)
Fifth	2.1% (0.68–5.7%)	5.1% (3.5–7.6%)	14% (11–17%)	13% (7.7–20%)
*n*	911	2321	2816	667

## Discussion

This study is the first to demonstrate that genetic risk scores can improve the selection of men for suspected prostate cancer investigation in primary care, over and above presenting clinical features. NICE guidance NG12 proposes that any combination of clinical features that represent a ≥3% chance of cancer should be investigated [11], although a reduction to 2% is under consideration to improve cancer outcomes [27]. The integrated risk model presented in this study could be used to risk stratify men with LUTS above and below this threshold. All individuals in the lower 3 quintiles (60% of men in UKBB with symptoms) could potentially be managed in primary care, avoiding referral. Individuals in the lower 2 quintiles of GRS (40%) could avoid referral under the proposed 2% threshold. Using the proposed 2% threshold, the integrated risk model suggests excluding GRS quintiles 1–4 in those aged under 60 years and quintile 1 in those aged 60–70 years.

### Limitations

This analysis was limited to white European ancestry due to the lack of ethnic diversity in UKBB; a substantial limitation as black men are twice as likely to be diagnosed with, and suffer worse outcomes from, prostate cancer [31]. As recruitment occurred between 2006 and 2010 when the men were aged 40–70 years, the cohort is enriched with younger men. This may result in an overestimate of the power of GRS if it is stronger at identifying prostate cancer in younger men; Conti et al.’s GRS was significantly associated with younger age at diagnosis [14]. However, this could also result in an underestimate of the true predictive value of GRS in symptomatic men. This study examines men in UKBB with a code for LUTS, which may not represent all men seeing their GP with such symptoms. There is also a lack of standardised follow-up across the cohort. The UK Biobank’s cancer registry data contains only diagnosis data from HES records and GP records, precluding us from studying tumour aggressiveness. A complete model of genetic susceptibility to prostate cancer would further include high-penetrance rare variants, which are not included in the selected GRS.

### Comparison to the existing literature

The performance of the integrated risk model is similar to the diagnostic accuracy of PSA as reported in the literature: AUC 0.72 (95% CI 0.68–0.76) [12]. We hypothesise that the optimal predictive model would incorporate PSA, GRS, and other clinical features. Oto et al.’s model achieved AUC of 0.71 (95% CI: 0.67–0.75) combining total PSA, free PSA, and age as predictors [32], although only total PSA is available in UK primary care. Seibert et al. developed a model that predicted age at onset of prostate cancer in men enrolled in the PROTECT trial to a high degree of accuracy in their validation study (*z* = 15.4, *P* < 10^−16^) [33]. That trial focussed on screening, rather than symptomatic detection, but also found that family history of prostate cancer added little predictive value. In that study, PSA was more predictive of prostate cancer in increasing centiles of risk score. Further research is needed to determine the best way to combine GRS with existing triage tools available in primary care, such as the PSA test, and to externally validate integrated risk models. Identifying aggressive prostate cancer is a key focus of prostate cancer diagnosis research efforts; this could not be assessed in the present study due to a lack of cancer stage data. About half of men with aggressive prostate cancer in Conti et al.’s study had a GRS in the top 20% [14].

### Clinical implications

This work has significant implications for the suspected prostate cancer investigation pathway in UK primary care. With the integration of GRS into routine clinical care, men identified as being at the greatest risk of prostate cancer could be prioritised for investigation, resulting in expedited diagnosis. The best available evidence supports the position that cancer diagnosis at an earlier disease stage is beneficial for survival [34]. Conversely, those identified as being at a very low risk of cancer by the integrated risk model could be managed in primary care and avoid invasive investigations, reducing patient harm, and reducing demand on secondary care services.

The ideal place for an integrated risk model in primary care would be as stratification tool to support GP decision-making for patients with LUTS, perhaps in deciding when to offer a PSA test. We have shown that, for prostate cancer, 40% of men with LUTS could avoid investigation for suspected cancer. Genetic sequencing is not currently available in UK primary care but current trends suggest that it will become part of routine practice in the future. The NHS will be the first national health care system to offer whole genome sequencing as part of routine care [35]. The NHS Genomic Medicine Service has included the use of GRS as a key area of interest [36] and programmes such as Our Future Health [37] will facilitate the translation of GRS studies in the future. The present study supports that development and shows for the first time that the availability of genomic data in primary care could benefit men with LUTS, although further research to consider patient preferences for genomic testing will be vital. Our integrated risk model approach could be applied using published GRS for other cancer types across multiple suspected cancer pathways; this has the potential to improve the investigation of symptomatic patients in primary care.

### Reporting summary

Further information on research design is available in the Nature Research Reporting Summary linked to this article.

## Supplementary information

Supplementary material

Reporting summary checklist

STROBE checklist

## Data Availability

All data in this project were part of the UK Biobank resource and was accessed under application number 74981. Information on how to access the UK Biobank can be found at https://www.ukbiobank.ac.uk/enable-your-research/apply-for-access.

## Code availability

All code used to generate results for this study can be found on the author’s Github page: https://github.com/hdg204/ProstateCancer.

## References

[1] Cancer Research UK. Prostate cancer incidence statistics [Internet]. [cited 2021 Feb 15]. https://www.cancerresearchuk.org/health-professional/cancer-statistics/statistics-by-cancer-type/prostate-cancer/incidence

[2] Abdel-Rahman M, Stockton D, Rachet B, Hakulinen T, Coleman MP (2009). What if cancer survival in Britain were the same as in Europe: how many deaths are avoidable?. Br J Cancer.

[3] McPhail S, Johnson S, Greenberg D, Peake M, Rous B. Stage at diagnosis and early mortality from cancer in England. Br J Cancer. 2015;112:S108–15. 10.1038/bjc.2015.49 PMC4385983 25734389

[4] Hamilton W. Cancer diagnosis in primary care. Br J General Practice. 2010;60:121–8. 10.3399/bjgp10X483175 PMC2814263 20132704

[5] Schröder FH, Hugosson J, Roobol MJ, Tammela TL, Zappa M, Nelen V (2014). The European randomized study of screening for prostate cancer-prostate cancer mortality at 13 years of follow-up HHS public access. Lancet..

[6] Martin RM, Donovan JL, Turner EL, Metcalfe C, Young GJ, Walsh EI (2018). Effect of a low-intensity PSA-based screening intervention on prostate cancer mortality: The CAP randomized clinical trial. J Am Med Assoc.

[7] Østerø Í Jákupsstovu J, Brodersen J. Do men with lower urinary tract symptoms have an increased risk of advanced prostate cancer? BMJ. 2018;361:k1202. 10.1136/bmj.k1202 29724877

[8] Marcus PM, Prorok PC, Miller AB, DeVoto EJ, Kramer BS (2015). Conceptualizing overdiagnosis in cancer screening. J Natl Cancer Inst.

[9] Just J, Osgun F, Knight C. Lower urinary tract symptoms and prostate cancer: is PSA testing in men with symptoms wise? Br J General Practice; 2018;68:541–2. 10.3399/bjgp18X699689 PMC6193782 30361318

[10] Hamilton W, Sharp DJ, Peters TJ, Round AP. Clinical features of prostate cancer before diagnosis: a population-based, case-control study. Br J General Practice. 2006;56:756–62. PMC1920715 17007705

[11] NICE: National Institute for Health and Care Excellence. Suspected cancer: recognition and referral. Ng12. 2017; https://www.nice.org.uk/guidance/ng12

[12] Merriel SWD, Pocock L, Gilbert E, Creavin S, Walter FM, Spencer A, et al. Systematic review and meta-analysis of the diagnostic accuracy of prostate-specific antigen (PSA) for the detection of prostate cancer in symptomatic patients. BMC Med. 2022;20:54. 10.1186/s12916-021-02230-y PMC8819971 35125113

[13] Usher-Smith JA, Sharp SJ, Griffin SJ. The spectrum effect in tests for risk prediction, screening, and diagnosis. BMJ 2016;353:i3139. 10.1136/bmj.i3139 PMC4916916 27334281

[14] Conti D v, Darst BF, Moss LC, Saunders EJ, Sheng X, Chou A, et al. Trans-ancestry genome-wide association meta-analysis of prostate cancer identifies new susceptibility loci and informs genetic risk prediction. Nat Genet. 2021;53:65–75. 10.1038/s41588-020-00748-0 PMC8148035 33398198

[15] Rashkin SR, Graff RE, Kachuri L, Thai KK, Alexeeff SE, Blatchins MA, et al. Pan-cancer study detects genetic risk variants and shared genetic basis in two large cohorts. Nat Commun. 2020;11:1–14. 10.1038/s41467-020-18246-6 PMC7473862 32887889

[16] Fantus RJ, Helfand BT. Germline genetics of prostate cancer: time to incorporate genetics into early detection tools. Clin Chem. 2019;65:74–9. 10.1373/clinchem.2018.286658 30459162

[17] Helfand B, Kearns J, Conran C, Xu J (2016). Clinical validity and utility of genetic risk scores in prostate cancer. Asian J Androl.

[18] Lewis C, Vassos E (2020). Polygenic risk scores: from research tools to clinical instruments. Genom Med.

[19] Manichaikul A, Mychaleckyj J, Rich S, Daly K, Sale M, Chen W (2010). Robust relationship inference in genome-wide association studies. Bioinformatics..

[20] Hamilton W, Lancashire R, Sharp D, Peters TJ, Cheng KK, Marshall T. The importance of anaemia in diagnosing colorectal cancer: a case–control study using electronic primary care records. Br J Cancer. 2008;98:323–7. 10.1038/sj.bjc.6604165 PMC2361444 18219289

[21] Bailey SER, Ukoumunne OC, Shephard EA, Hamilton W (2017). Clinical relevance of thrombocytosis in primary care: a prospective cohort study of cancer incidence using UK electronic medical records and cancer registry data. Br J Gen Pract.

[22] Stapley S, Peters TJ, Neal RD, Rose PW, Walter FM, Hamilton W (2013). The risk of oesophago-gastric cancer in symptomatic patients in primary care: A large case-control study using electronic records. Br J Cancer.

[23] Shephard EA, Stapley S, Neal RD, Rose P, Walter FM, Hamilton W (2012). Clinical features of bladder cancer in primary care. Br J Gen Pract.

[24] Hopkins R, Bailey SER, Hamilton WT, Shephard EA (2020). Microcytosis as a risk marker of cancer in primary care: A cohort study using electronic patient records. Br J Gen Pract.

[25] Shephard E, Neal R, Rose P, Walter F, Hamilton W (2013). Clinical features of kidney cancer in primary care: a case-control study using primary care records. Br J Gen Pract.

[26] Price SJ, Shephard EA, Stapley SA, Barraclough K, Hamilton WT (2014). Non-visible versus visible haematuria and bladder cancer risk: a study of electronic records in primary care. Br J Gen Pract.

[27] Moore SF, Price SJ, Chowienczyk S, Bostock J, Hamilton W. The impact of changing risk thresholds on the number of people in England eligible for urgent investigation for possible cancer: an observational cross-sectional study. Br J Cancer. 2021;125:1593–7. 10.1038/s41416-021-01541-4 PMC8445014 34531548

[28] Banks J, Hollinghurst S, Bigwood L, Peters TJ, Walter FM, Hamilton W. Preferences for cancer investigation: a vignette-based study of primary-care attendees. Lancet Oncol. 2014;15:232–40. 10.1016/S1470-2045(13)70588-6 24433682

[29] von Elm E, Altman DG, Egger M, Pocock SJ, Gotzsche PC, Vandenbroucke JP (2007). The strengthening the reporting of observational studies in epidemiology (STROBE) statement: guidelines for reporting observational studies. Ann Intern Med.

[30] Green HD, Merriel SWD, Oram RA, Ruth KS, Tyrrell J, Jones SE, et al. Applying a genetic risk score for prostate cancer to men withlower urinary tract symptoms in primary care to predict prostate cancerdiagnosis: a cohort study in the UK Biobank. 2022. Preprint at https://www.medrxiv.org/content/10.1101/2022.01.21.22269629v1. 10.1038/s41416-022-01918-z PMC9553867 35978138

[31] Hoffman RM, Frank D, Eley JW, Linda C, Stephenson RA, Stanford L (2001). Racial and ethnic differences in advanced-stage prostate cancer: the prostate cancer outcomes study. J Natl Cancer Inst.

[32] Oto J, Fernández-Pardo Á, Royo M, Hervás D, Martos L, Vera-Donoso CD, et al. A predictive model for prostate cancer incorporating PSA molecular forms and age. Sci Rep. 2020;10:1–10. 10.1038/s41598-020-58836-4 PMC7016114 32051423

[33] Seibert TM, Fan CC, Wang Y, Zuber V, Karunamuni R, Parsons JK (2018). Polygenic hazard score to guide screening for aggressive prostate cancer: development and validation in large scale cohorts. BMJ.

[34] Neal RD, Tharmanathan P, France B, Din NU, Cotton S, Fallon-Ferguson J (2015). Is increased time to diagnosis and treatment in symptomatic cancer associated with poorer outcomes? Systematic review. Br J Cancer.

[35] Alderwick H, Dixon J. The NHS long term plan. BMJ. 2019;364:l84. 10.1136/bmj.l84 PMC6350418 30617185

[36] Genomics Education Programme. NHS launches new polygenic scores trial for heart disease [Internet]. 2021. https://www.genomicseducation.hee.nhs.uk/blog/nhs-launches-new-polygenic-scores-trial-for-heart-disease/

[37] Our Future Health [Internet]. 2022. https://ourfuturehealth.org.uk/

[38] Green H, Jones A, Evans J, Wood A, Beaumont R, Tyrrell J (2021). A genome-wide association study identifies 5 loci associated with frozen shoulder and implicates diabetes as a causal risk factor. PLoS Genet.

