# Supplementary material for: Applying a genetic risk score for prostate cancer to men with lower urinary tract symptoms in primary care to predict prostate cancer diagnosis: a cohort study in the UK Biobank
Source: Br J Cancer. 2022 Aug 18;127(8):1534–9. doi: 10.1038/s41416-022-01918-z (PMC9553867; doi:10.1038/s41416-022-01918-z)
Supplement: Supplementary file 1 — Supplementary material [file 41416_2022_1918_MOESM1_ESM.docx]

## Supplementary Table 1

Read 3 codes used to define prostate cancer and symptoms. Prostate cancer codes were used to define case / control status. All other codes were used as inclusion criteria.

| read_3 | category |
| --- | --- |
| 1A27. | DoubleVoiding |
| K16y8 | DoubleVoiding |
| XaNFc | DoubleVoiding |
| X30Ni | DoubleVoiding |
| 1A1Z. | Frequency |
| 1A11. | Frequency |
| 1A1.. | Frequency |
| R084. | Frequency |
| 1A12. | Frequency |
| R084z | Frequency |
| R0840 | Frequency |
| 1A1.. | Frequency |
| 1A1.. | Frequency |
| 1A34. | Hesitancy |
| 1A34. | Hesitancy |
| R083z | Incontinence |
| R083. | Incontinence |
| 1AZ6. | LUTS |
| 1AZ60 | LUTS |
| 1AZ61 | LUTS |
| 1AZ62 | LUTS |
| 1A2.. | LUTS |
| 1A2Z. | LUTS |
| R086z | LUTS |
| 8D7.. | LUTS |
| R08.. | LUTS |
| R08zz | LUTS |
| 66K3. | LUTS |
| 1A4.. | LUTS |
| 1A… | LUTS |
| 1A… | LUTS |
| 1AZ.. | LUTS |
| 1AZZ. | LUTS |
| 1AH1. | LUTS |
| R086. | LUTS |
| R08z. | LUTS |
| Kz… | LUTS |
| Ryu4. | LUTS |
| XaB9O | LUTS |
| XaXHi | LUTS |
| XaXHj | LUTS |
| XaXHk | LUTS |
| Xa96j | LUTS |
| 1A13. | Nocturia |
| R0842 | Nocturia |
| 1A33. | PoorStream |
| 1A31. | PoorStream |
| 1A3.. | PoorStream |
| 1A3Z. | PoorStream |
| 1A3.. | PoorStream |
| R0861 | PoorStream |
| R0863 | PoorStream |
| R0860 | PoorStream |
| 317C. | PoorStream |
| 1A37. | PoorStream |
| 1A36. | PoorStream |
| XaD2w | PoorStream |
| X77SF | PoorStream |
| X76Y0 | PoorStream |
| R082. | Retention |
| R0824 | Retention |
| 1A32. | Retention |
| K196. | Retention |
| R0820 | Retention |
| 1A32. | Retention |
| R0822 | Retention |
| 1A25. | Urgency |
| R0862 | Urgency |
| 1A25. | Urgency |
| R15y0 | ProstateCancer |
| B7C20 | ProstateCancer |
| 14270 | ProstateCancer |
| ZV104 | ProstateCancer |
| B834. | ProstateCancer |
| 1J08. | ProstateCancer |
| B58y5 | ProstateCancer |
| B8340 | ProstateCancer |
| B46.. | ProstateCancer |
| XaC0j | ProstateCancer |
| Xa3fu | ProstateCancer |
| XaXGk | ProstateCancer |
| XaFwo | ProstateCancer |
| XaKyV | ProstateCancer |

## Supplementary Table 2

Number of people included broken down by which symptom they were included for. Note that as it is possible for an individual to report two symptoms on the index date, n sums to more than the cohort total and percent sums to more than 100.

| symptoms | n | percent |
| --- | --- | --- |
| LUTS | 1629 | 24.0 |
| Incontinence | 26 | 0.4 |
| Nocturia | 1686 | 24.9 |
| Hesitancy | 164 | 2.4 |
| Frequency | 2001 | 29.5 |
| Urgency | 580 | 8.6 |
| Retention | 363 | 5.4 |
| PoorStream | 678 | 10.0 |
| DoubleVoiding | 13 | 0.2 |

## Supplementary Table 3

Area Under the Curve (AUC) statistics of all permutations of GRS, age, family history (FH) and symptom profile.

| modelname | aucs | cis |
| --- | --- | --- |
| GRS | 0.703 | 0.67-0.736 |
| age | 0.680 | 0.65-0.709 |
| FH | 0.514 | 0.495-0.533 |
| Symptoms | 0.609 | 0.573-0.644 |
| GRS+age | 0.772 | 0.744-0.8 |
| GRS+FH | 0.704 | 0.671-0.737 |
| GRS+Symptoms | 0.729 | 0.698-0.76 |
| age+FH | 0.684 | 0.655-0.714 |
| age+Symptoms | 0.700 | 0.671-0.728 |
| FH+Symptoms | 0.609 | 0.573-0.646 |
| GRS+age+FH | 0.773 | 0.745-0.801 |
| GRS+age+symptoms | 0.781 | 0.754-0.809 |
| GRS+FH+Symptoms | 0.730 | 0.699-0.761 |
| age+FH+symptoms | 0.703 | 0.674-0.732 |
| GRS+age+FH+symptoms | 0.782 | 0.755-0.81 |

## Supplementary Figure 1


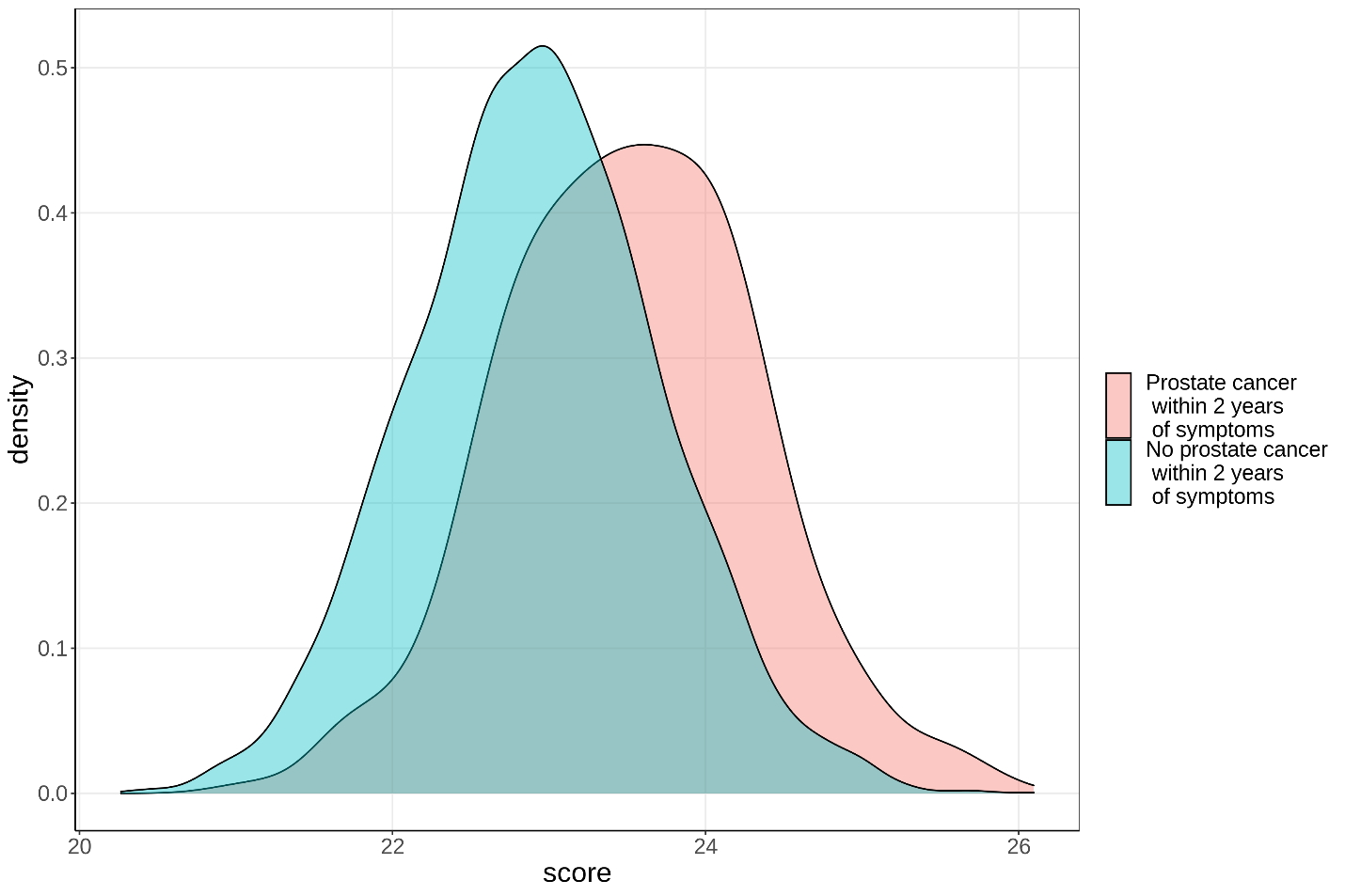


Supplementary Figure 1 - Density plot showing the distributions in the genetic risk score of those who were diagnosed with cancer within 2 years of symptoms (red) and those who were not (blue).

## Supplementary Figure 2


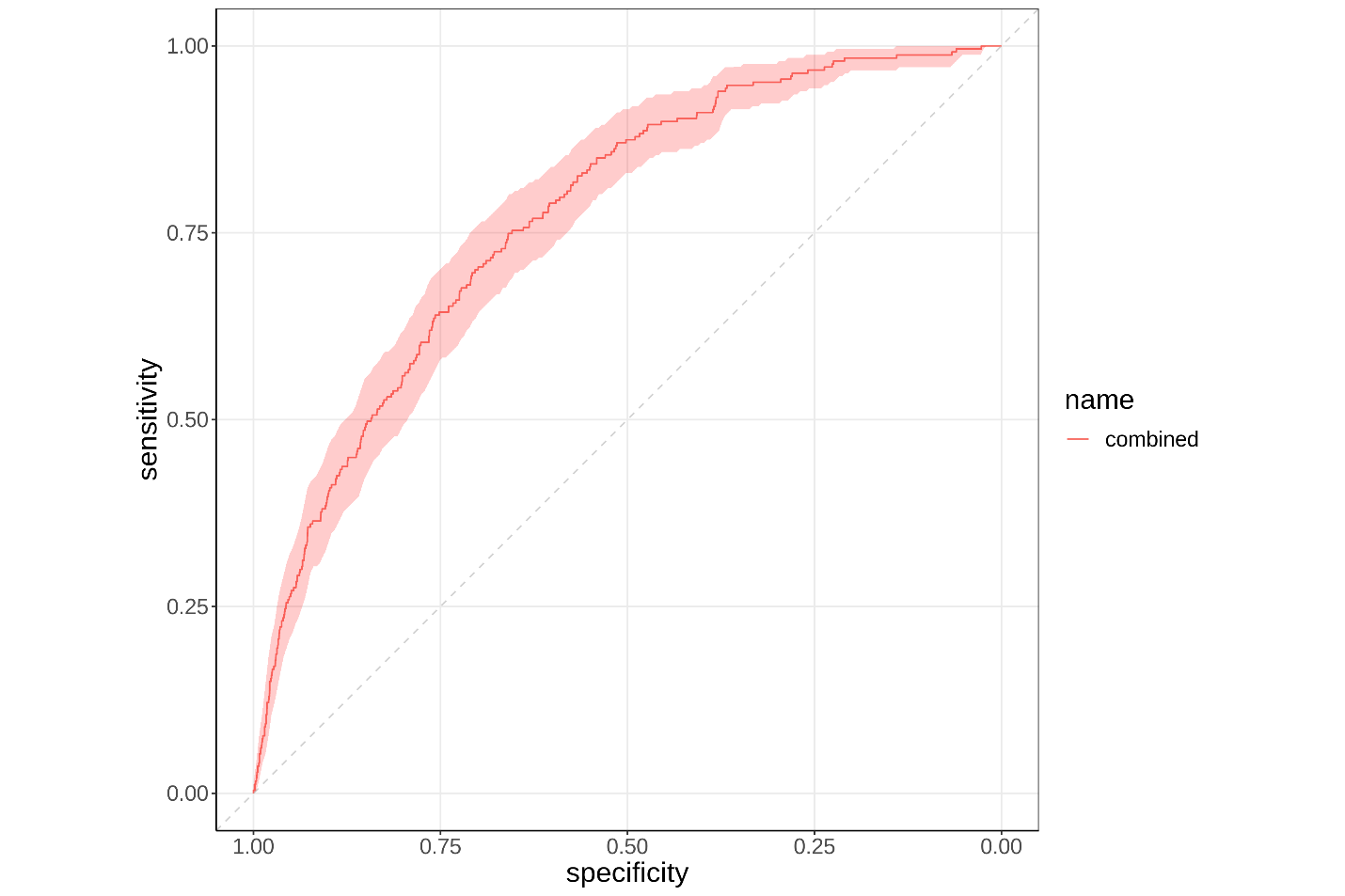


Supplementary Figure 2 - ROC curve of an integrated risk model including GRS and age to predict prostate cancer within 2 years of symptoms. The ROC AUC was 0.768 (95% CI 0.739 to 0.796)
